# Empathy study Cologne: A cross-sectional analysis exploring empathy in German dental students

**DOI:** 10.3205/zma001811

**Published:** 2026-02-17

**Authors:** Isabel Deeg, Constantin Justus Serwe, Nils Spiekermann, Evamarie Brock-Midding, Nicolas Frenzel Baudisch, Christoph Matthias Schoppmeier, Anna Greta Barbe, Michael Jochen Wicht

**Affiliations:** 1 University of Cologne, Faculty of Medicine and University Hospital Cologne, Polyclinic for Operative Dentistry and Periodontology, Cologne, Germany; 2 Institut der Deutschen Zahnärzte (IDZ), Cologne, Germany; 3 infas Institute of Applied Social Science GmbH, Bonn, Germany

**Keywords:** empathy, dental education, physician-patient relations, patient-centered dentistry

## Abstract

**Objectives:**

Empathy is the basis for patient-centered dentistry, but there are gaps in understanding empathy development within dental education. This study aimed to assess empathy levels among German dental students, including influencing factors such as gender, study year, age, and career interests.

**Methods:**

This cross-sectional study was conducted during the summer term in 2023, involving 155 dental students from the University of Cologne, Germany. Empathy levels were measured using the Jefferson Scale of Empathy-Health Professions Students (JSE-HPS) version (twenty items, 7-point-Likert scale, range 20-140 points). Sociodemographic data, including age, gender, study year, and career aspirations, were collected for each student and analyzed.

**Results:**

On average German dental students exhibited high empathy scores: 110.20±11.81 (standard deviation) (min 68.00, max 136.00). Significant differences were observed based on gender, with female students scoring higher on the JSE-HPS than their male counterparts (*p*<0.0001, Cohen’s d=0.92). A notable decrease in empathy was found among male students in the final two years of study (years three to four, p=0.005; years three to five, *p*=0.004). No significant correlations were observed between empathy levels and student age (*p*=0.074) or career interests (*p*=0.593).

**Conclusions:**

High levels of empathy among German dental students were observed, associated with gender specific differences and a significant decrease in empathy in males over the last two years of study. These findings highlight the need for targeted educational interventions, especially in the later years of study, to maintain and promote empathy throughout dental education.

## Zusammenfassung

**Ziele:**

Empathie bildet die Grundlage patientenzentrierter Zahnmedizin, jedoch bestehen Wissenslücken hinsichtlich der Entwicklung von Empathie innerhalb der zahnmedizinischen Ausbildung. Ziel dieser Studie war es, das Empathieniveau deutscher Zahnmedizinstudierender zu erheben, einschließlich von Einflussfaktoren wie Geschlecht, Studienjahr, Alter und späteren Berufswünschen.

**Methoden:**

Die Querschnittsstudie wurde im Sommersemester 2023 mit 155 Zahnmedizinstudierenden der Universität zu Köln durchgeführt. Die Empathieniveaus wurden mit der *Jefferson Scale of Empathy – Health Professions Students (JSE-HPS)* Version erhoben (20 Fragen, 7-Punkte-Likert-Skala, Wertebereich 20–140 Punkte). Soziodemografische Daten wie Alter, Geschlecht, Studienjahr und Berufswünsche wurden für jede teilnehmende Person erfasst und analysiert.

**Ergebnisse:**

Im Durchschnitt zeigten die Zahnmedizinstudierenden hohe Empathiewerte: 110,20±11,81 (Standardabweichung) (Minimum 68,00, Maximum 136,00). Signifikante Unterschiede zeigten sich zwischen den Geschlechtern: weibliche Studierende erzielten höhere Werte auf der JSE-HPS als ihre männlichen Kommilitonen (p<0,0001, Cohen’s d=0,92). Ein deutlicher Rückgang der Empathiewerte wurde bei männlichen Studierenden in den letzten beiden Studienjahren festgestellt (Jahr 3-4: p=0,005; Jahr 3-5: p=0,004). Zwischen Empathieniveau und Alter (p=0,074) oder späteren Berufswünschen (p=0,593) bestanden keine signifikanten Zusammenhänge.

**Schlussfolgerungen:**

Die Untersuchung zeigte insgesamt hohe Empathiewerte unter deutschen Zahnmedizinstudierenden, wobei sich geschlechtsspezifische Unterschiede sowie ein signifikanter Rückgang der Empathiewerte bei männlichen Studierenden in den letzten beiden Studienjahren abzeichneten. Diese Befunde betonen die Notwendigkeit gezielter curricularer Maßnahmen, insbesondere in den späteren Ausbildungsabschnitten, um Empathie langfristig zu bewahren und weiterzuentwickeln.

## 1. Introduction

Participative doctor-patient communication is a key factor for concordant treatment decisions and leads to better clinical outcomes and patient satisfaction. To generate a participative interaction between physicians and patients, physician empathy plays an important role [1]. Patients expect their physicians to be empathetic; in return, they exhibit greater cooperation, satisfaction, and concordance, leading to positive effects on health outcomes [2]. Physicians themselves also benefit from an ability to treat patients with empathy. Empathetic medical care leads to higher job satisfaction and helps to prevent burnout [3]. A lack of empathy has been associated with a higher likelihood of medical malpractice; depersonalization and emotional exhaustion in doctors led to a 54% higher risk of committing a medical error [4]. Empathy-centered doctor–patient communication is also beneficial for the healthcare system, with links to lower healthcare utilization, a reduction in unnecessary office visits, fewer referrals to other specialties, and fewer imaging and diagnostic procedures [5], [6]. 

In dentistry, empathy fosters trust between dentists and patients, reduces anxiety, and enhances patient autonomy by creating a respectful and supportive treatment environment [7]. In the last decade, modern dental education has not only prioritized the development of clinical skills, but also the importance of patient-centered care, with a focus on strong communication skills – including student empathy [8]. However, the empathy levels of dental students reported globally are heterogeneous. For example, Polish dental students reached their empathy peak in study year four; in contrast, first-year students had the highest empathy levels in British and US studies [9], [10], [11]. Comparative, cross-sectional studies have demonstrated that in the US, female osteopathic medical students were more emphatic than their male peers, and that empathy levels decreased significantly over the course of their studies [12]. In the area of dentistry, according to data retrieved from a systematic review [13], some studies found that male dental students were slightly – but often non-significantly – more empathetic than females, while others reported the opposite (see table 1 (Tab. 1)) [11], [14], [15], [16], [17], [18], [19]. However, the majority of medical and dental empirical research has found that women are more empathetic than their male counterparts [13], [20]. 

Since their introduction in 2020, German regulations for dental courses have enhanced empathy-influencing interventions to foster fundamental values, standards, and consideration of the fundamental ethical principles and core values of dental and medical practice [21], [https://www.gesetze-im-internet.de/zappro/BJNR093310019.html]. However, the actual level of empathy in German dental students remains largely unexplored. A 2022 study at the University of Frankfurt/Main, Germany, using the JSPE-S found no gender differences in empathy levels in the 2^nd^ and 4^th^ year, but comprehensive data across all five years are lacking [22]. This knowledge is important to enable the dental curriculum to be implemented and adjusted to meet the necessary criteria, and to ensure that students can display a high standard of empathy when graduating and become professional, empathy-trained dentists.

We hypothesize that the level of empathy among dental students declines over the course of their studies, particularly as clinical training increasingly shifts toward operative and technically demanding procedures on patients [11]. It is essential to determine whether, when, and under which conditions (e.g., gender, age) empathy levels in dental students change throughout their studies. Only with this knowledge can targeted interventions – such as simulated role plays or communication skills training [23] – be implemented effectively and at the most appropriate stage of the curriculum.

Therefore, this cross-sectional study (summer term 2023) aimed to investigate empathy levels in dental students from curricular years one to five, as well as potential influencing factors such as age, gender, and career aspirations.

## 2. Material and methods

The study was approved by the local ethics committee (22-1225-1; October 31, 2022) and registered at the German Register of Clinical Studies (DRKS00032649). The study was conducted in accordance with the principles of the Declaration of Helsinki, and all participants provided written informed consent prior to their inclusion.

### 2.1. Participants and data collection

All participants were enrolled from the Faculty of Dentistry within the ten semesters of the dental, oral, and maxillofacial studies at the University of Cologne, Germany, from June 2023. Four hundred students were invited to voluntarily participate and complete the questionnaire via email. Data collection was performed using the web-based questionnaire tool SurveyMonkey^®^ [24]. All study data were pseudonymized in accordance with data protection regulations prior to analysis and the data provided could not be traced back to participants. Before completing the survey, participants received a study information email asking for informed consent. Consent was given online by submission of the declaration of consent and completion of the questionnaire. 

### 2.2. Questionnaire design 

The Jefferson Scale of Empathy (JSE) is a twenty-item instrument designed to measure empathy in health professionals. Of the three different versions available [25], and as recommended by Thomas Jefferson University, the JSE for health professional students (JSE-HPS) was chosen as it was most suited to the study population [26]. 

The JSE-HPS questionnaire consists of two parts. Part 1 was expanded to include questions related to the sociodemographic background of participants, including age, gender, semester, aspired professional orientation, and ethnic parameters such as place of birth, place of growing up, and language skills (native language). Part 2 consists of twenty questions for each target group, with response options in the form of a seven-point Likert scale (1=strongly disagree to 7=strongly agree). Half of the items (1, 3, 6, 7, 8, 11, 12, 14, 18, 19) constitute “reverse scoring”. Total scores on the JSE-HPS range from 20 to 140, with higher scores indicating higher levels of empathy [25]. The scale was translated into German, using the back translation method outlined below [27]. The questionnaire also contained an antecedent information section that included privacy information, conditions of participation, design, and study purpose.

### 2.3. Pilot study

To generate a validated German version of the JSE-HPS, the English questionnaire was translated into German by two native German speakers using the “back translation method”; this was then translated back into English by two native English speakers [27]. To identify any additional language difficulties, ensuring correct interpretation of the questions and check of the time needed to complete the questionnaire, the questionnaire was send to eighteen dental professionals from the Faculty of Medicine at the University of Cologne, Germany, in a pilot study [26], [27]. The pilot participants completed the questionnaire (German version of the JSE-HPS) in approximately 10 min. No misinterpretations were identified in answering the questions (Cronbach’s α=0.60).

### 2.4. Statistical analysis

Statistical analyses were conducted using the IBM SPSS Statistics software package (IBM Corp. Released 2023. IBM SPSS Statistics for Macintosh, Version 29.0.1. Armonk, NY: IBM Corp.). All participants who fully completed the JSE-HPS questionnaire were considered in the final analysis (per-protocol analysis). The significance level was set at α=0.05 (*p*<0.05). Descriptive statistics included frequencies (in percentages), key figures of the central tendency (average mean values), as well as measures of variability (minimum and maximum values, standard deviations (SD)). ANOVA was conducted using analyses of variances, univariable linear regression analysis, and t-tests for independent samples. A multivariable linear regression analysis was performed to determine the factors that correlated independently with empathy scores. Reverse coding was used for the ten negatively worded items of the JSE-HPS. The total score was calculated for each participant by summing up the scores of the responses to the twenty items of the JSE-HPS scale. 

## 3. Results

### 3.1. Response rate and sample characteristics

**Figure 1:**
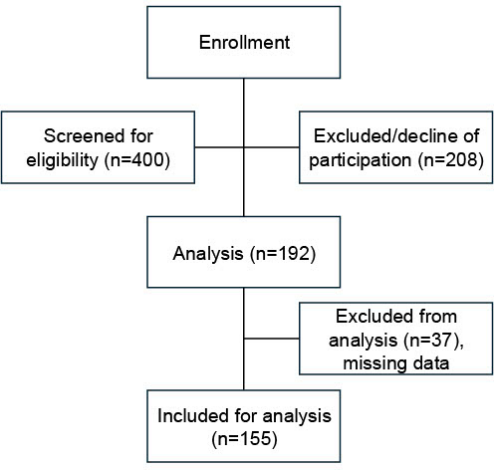
Study flow chart

In total, n=192 students completed the questionnaire; thirty-seven of these surveys were not included due to missing information (see figure 1 (Fig. 1)). Thus, full data was available for n=155 students, and all gave written consent by submitting the completed survey. Students were distributed evenly across all semesters of the program. 

Overall, 104/155 (67.1%) participants were female, fifty (32.3%) were male, and one (0.6%) classed themselves as “other” (see table 2 (Tab. 2)). When asked about their dental career aspirations, forty-three (27.74%) participants chose a “procedure-orientated” specialty (orofacial surgery), seventy-one (45.8%) preferred a “people-orientated” specialty and forty-one (26.5%) were undecided. Most participants (ninety-six; 61.9%) were younger than 24 years (i.e., the usual age to finish dental studies without any delays).

### 3.2. JSE scores according to year of study, gender, age, and professional aspirations

**Figure 2:**
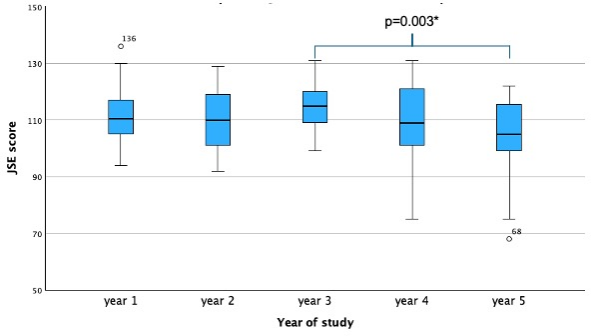
Boxplot of empathy scores (Jefferson Scale of Empathy, JSE) by year of study; a significant difference is evident between year three and year five students

The mean overall JSE score was 110.20 (SD 11.81; min 68.00, max 136.00; percentiles: 5^th^=89.40, 25^th^=104.00, 50^th^ (median)=111.00, 75^th^=118.00, 95^th^=128.20). ANOVA showed an overall difference between the five study years (*p*=0.009) with a significant difference between years three and five (*p*=0.003) (see figure 2 (Fig. 2)). No significant differences in empathy levels were detected between other study years. Regression analysis showed a decline in empathy scores as students progressed during their studies (*p*=0.042). 

The t-test resulted in a difference in JSE scores between males and females (*p*<0.0001, Cohen’s d=0.92). Univariate linear regression analysis showed a difference in JSE scores in favor of females (*p*<0.0001). Over the five years of study, ANOVA showed no difference in JSE scores for females (*p*=0.632) whereas there was a significant reduction in male students (*p*=0.002). The post hoc analysis found a significant difference for male students between years three and four (*p*=0.005) and years three and five (*p*=0.004), but no differences for females. 

Univariable linear regression analysis demonstrated a decline in JSE scores over the years of study, which was particularly evident among male students, whose empathy scores decreased over time (*p*=0.013). In contrast, female students did not exhibit changes in empathy levels across the five years of study (*p*=0.303).

There was no difference in JSE scores compared to participants’ age group (*p*=0.074), with a positive trend towards differences in JSE scores with increasing age (*p*=0.217). No difference in JSE scores could be observed according to their professional dental career aspirations (*p*=0.593)

## 4. Discussion

Dental students in Cologne, Germany, had high empathy scores with 50% of participants scoring above the calculated median of 111.00 as suggested in recent literature [28]. Slightly higher mean JSE scores have been found in US dental students [11], while other studies have reported lower mean empathy scores in dental students in Saudi Arabia [29], Nigeria [30], Pakistan [31], the UK [10], and in Poland and Croatia [32] (see table 3 (Tab. 3)). These findings may be explained by differences in culture, religious beliefs, and race and ethnicities as for example in Saudi Arabia dentistry used to be male dominated with a recent increase in female students, as dentistry became a well-accepted course to study for women [33] whereas in Europe or Northern America the gender gap in graduating dental students has been narrowed [34]. Recent empirical research from the US has reported higher JSE scores in African-American (117.08±11.50) and Hispanic/Latino/Spanish (116.56±11.74) medical students compared to Caucasian/white (115.34±12.08) or Asian (114.32±12.88) students [12]. Such findings may be explained by the so-called “wounded healer effect” [35], which suggests that individuals who have experienced suffering in terms of for example racism or personal illness are better able to empathize with others through shared experiences, particularly in the context of cultural minorities facing discrimination – this may enhance their empathy towards those in distress, such as dental patients [12], [36].

Another key result of this study was the significant decrease in empathy levels in students between years three and five, as well as years three and four. After year three, students at Cologne University finish theoretical preclinical learning and actively start supervised patient treatment. The decline may indicate that the shift from theoretical studies to hands-on patient care negatively impacts their empathy levels as they advance in their clinical education. Similar findings were observed in US dental and medical students [11], [37]. A possible explanation might be the difference between fictional expectations before engaging in patient care and experiences being a patient themselves versus the more dispassionate feelings towards patients when treating them clinically. The discrepancy between their initial expectations and real-life experience can contribute to this shift, as students may struggle with the emotional demands of patient care or become more focused on clinical efficiency and technical skills [38]. For example, Finish undergraduate male dental students stated to be more confident regarding upcoming clinical or technical procedures than their female counterparts [39]. 

Furthermore, research suggests an influence from the role models that students encounter during their training. These role models, whether intentionally or unintentionally, shape student attitudes towards patient care, with a focus on clinical efficiency – potentially leading to the neglect of empathetic practices. This dynamic highlights the importance of ensuring that role models in medical education balance the development of technical skills with the cultivation of empathy [40]. 

Another important finding in this study was that female students were overall more empathetic than males, with a decrease in JSE scores for males over the five years of study. Similar observations have been made in other studies in many different countries [11], [12], [29]. For example, when exposed to children crying or laughing, women were more sensitive towards infant crying than men (measured via brain imaging) [41]. These findings may be viewed as advantageous in patient care, as women often excel empathetically as caregivers. However, it is important to note that women in caregiving roles may also be more susceptible to stressful stimuli such as challenging working conditions compared to their male counterparts, which could pose a disadvantage in the medical field [42]. 

The decrease in empathy in this study was mainly observed in male dental students when entering their clinical study years. Although other research has shown that both female and male students display no differences in perceived stress levels during their studies, male dental students exhibit less emotional intelligence and perceive quality of life to be lower during their studies [43]. Overall stress levels are perceived as higher during their clinical years, which can be explained by the higher workload and more clinical placement hours [44]. For male students, an increase in stress levels might potentiate a lack of perceived quality of life and emotional intelligence when entering the clinical years, and therefore contribute towards the decrease in JSE scores [44], [45]. 

This study showed no significant differences with respect to the age of students and their JSE scores, with most participants being between 18-30 years old. However, there was a trend towards higher empathy levels in older students. These findings corroborate results for medical students [12]. Rather than age alone, the previous experience of students in the field of medicine (and hence a higher age when entering the study of dentistry) may influence empathy levels. Findings from a recent mixed-methods study with a population of dental students (unpublished data), as well as those from a study carried out with medical students [46], support this hypothesis.

Our study has some limitations. Only 192 out of 400 eligible dental students completed the JSE-HPS questionnaire. This may represent a potential bias, as students who are more willing to participate in such surveys might already exhibit higher levels of empathy compared to those who chose not to respond. The low response rate also raises questions about the chosen distribution method. Since the questionnaire was sent via email, a more personal or direct approach – such as distributing it during lectures or seminars – might have resulted in a higher return rate. This is particularly relevant given the small sample size in certain subgroups, such as second-year students (N=17). In future studies, it may be beneficial to use multiple recruitment channels and consider collecting additional variables, such as students’ motivation for participation or their workload at the time of the survey, to better understand participation patterns and potential biases. The study design used was cross-sectional during summer term 2023. It cannot be ignored that the observed differences in empathy levels between academic years may simply be due to the individual characteristics of the students in each semester. It is conceivable – although highly unlikely – that cohorts of higher academic years may include a disproportionate number of less empathetic male students, unrelated to the influence of the dental curriculum or clinical training. Without longitudinal data tracking individual trajectories of empathy development over multiple time points, it is impossible to make causal inferences about the specific role of dental education in shaping these trends. Overall, there was a decline in empathy levels from 111.00 to 104.14 over the years of the dentistry course. A possible explanation may the distribution of gender in two year-groups due to the cross-sectional study design: in the first year of study, 86% of participants were female in contrast to 43% in year five, with women being more empathetic than males. The overall decline in empathy during medical or dental education may also reflect a normal trajectory, as students develop professional detachment essential for patient care. Dentists often focus on technical details like radiographic analyses or probing depths, abstracting from patient symptoms. This shift does not imply a lack of attentiveness but reflects the demands of clinical practice. Female students may retain higher empathy levels compared to males, possibly due to social influences that reinforces relational and emotional labor, as previously discussed. This suggests that while declining empathy may be typical in training, gender-typical experiences could influence its extent. Moreover, the JSE-HPS only records a snapshot of the students’ level of empathy, which may depend, for example, on their personal daily perception of stress. Yet empathy goes far beyond a purely quantitative assessment and should be considered both on a qualitative and a quantitative basis. 

Empathy plays a crucial role in shaping the educational experience of dental students and the quality of care they will provide in the future. Empathy is not just a soft skill, but a key part of effective patient care in the context of patient-centered dentistry and shared decision-making. An empathetic dental student is more likely to engage in meaningful communication with patients, understand their needs and concerns, and actively involve them in their treatment plans. The ability to show empathy is essential in a modern dental practice, where the focus is increasingly on comprehensive and patient-centered approaches. Patients today are well informed and expect to be treated respectfully and to participate actively in treatment decisions. This shift requires dentists to be technically competent, but also capable of narrative dentistry. Empathy-enhancing interventions such as role-playing exercises, virtual patient simulations, communication skills training, and approaches from narrative dentistry should be implemented not during the early preclinical years, but rather toward the final stages of dental education, when empathy levels are more likely to decline. These methods allow students to actively engage with patients’ perspectives, reflect on their own communication styles, and strengthen their capacity for patient-centered care in complex clinical situations. For instance, training in shared decision making can foster empathy by encouraging students to actively involve patients in the treatment process, listen to their preferences and concerns, and collaboratively choose appropriate care options – skills that are particularly important in dentistry, where treatment often includes multiple viable alternatives. Future studies should therefore not only examine the effectiveness and design of such interventions, but also place particular emphasis on identifying the optimal timing for their implementation within the curriculum. Addressing a lack of empathy through targeted educational interventions and role modeling in dental schools could lead to future dentists who are not only skilled clinicians but also sensitive caregivers, with all the advantages of empathetic and compassionate care for patients, care providers, and ultimately the healthcare system.

## 5. Conclusion

This study shows generally high empathy scores among German dental students. Gender differences were significant, with female students showing higher empathy scores than males; the latter experienced a significant decrease in empathy scores in the final two years of study. Empathetic treatment of dental patients has the potential to improve treatment outcomes. Based on these results, future clinical interventions that influence the empathy level of students should be implemented in the later years of study to counteract the decline in empathy levels among students. It remains to be determined whether attempts to enhance empathy among dental students will be appreciated by patients in terms of general comfort, health literacy, self-efficacy, and ultimately improved treatment outcomes.

## Acknowledgements and funding

The draft manuscript was reviewed by a native English speaker, Deborah Nock (Medical WriteAway, Norwich, UK). The authors did not receive any funding for this work. Open access funding was facilitated and supported by ProjektDEAL.

## Authors’ ORCIDs

- Isabel Deeg: [0000-0003-0306-5873]
- Nils Spiekermann: [0000-0002-2329-1475]
- Evamarie Midding: [0000-0001-5198-2380]
- Nicolas Frenzel Baudisch: [0000-0003-2037-3747]
- Christoph Schoppmeier: [0000-0002-3269-8920]
- Anna Greta Barbe: [0000-0003-0169-2582]
- Michael J. Wicht: [0000-0003-4693-7184]

## Competing interests

The authors declare that they have no competing interests. 

## Figures and Tables

**Table 1:**
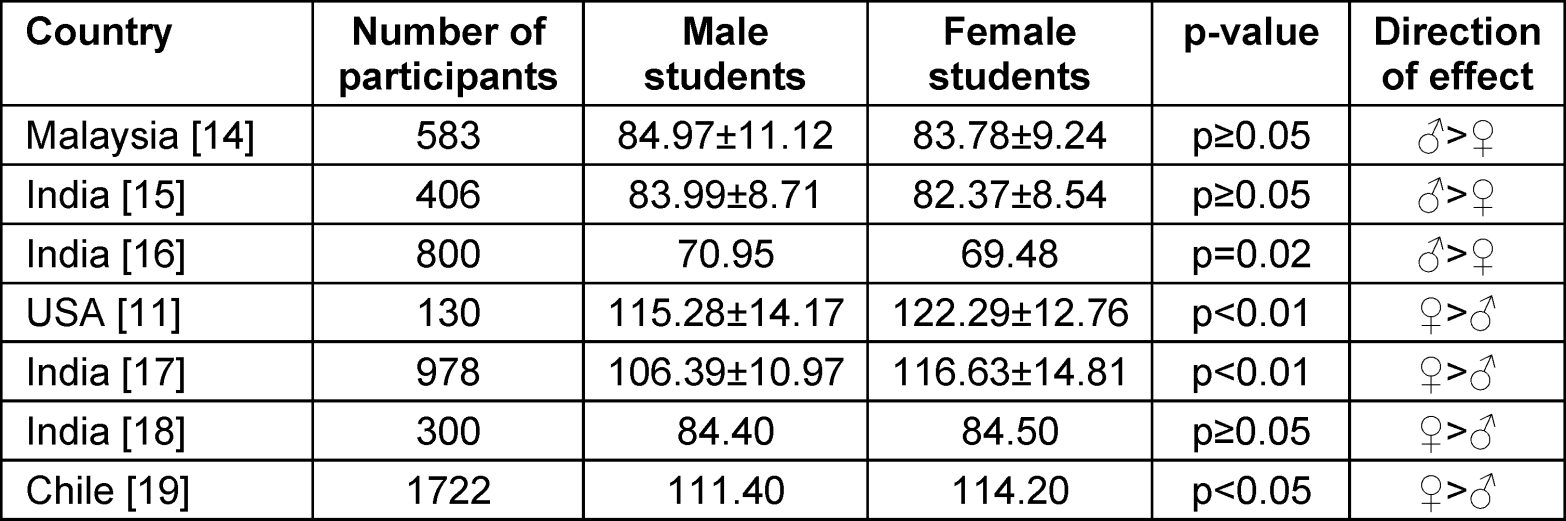
Empathy scores by gender in international dental student studies

**Table 2:**
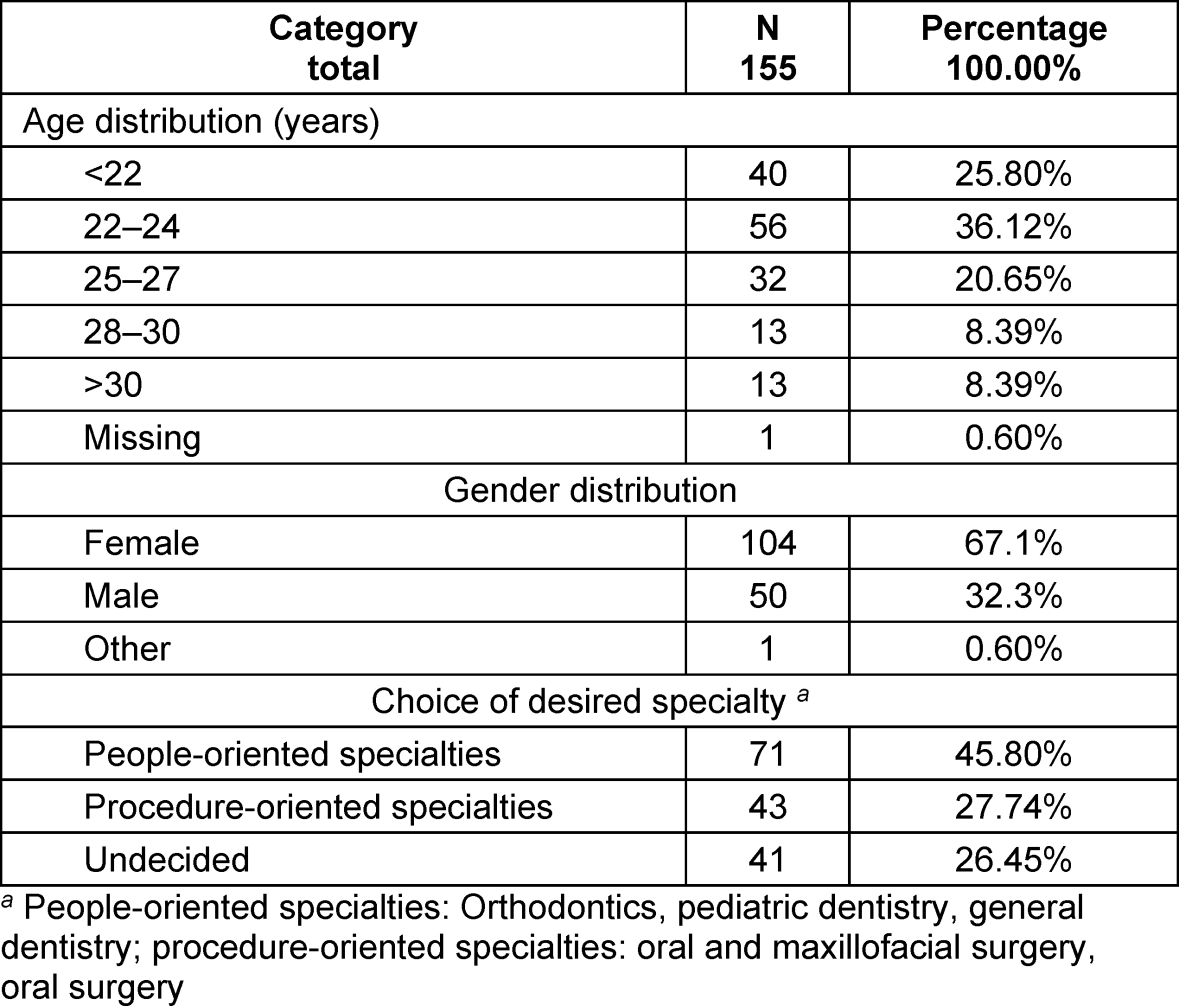
Characteristics of participants in the sample (N=155)

**Table 3:**
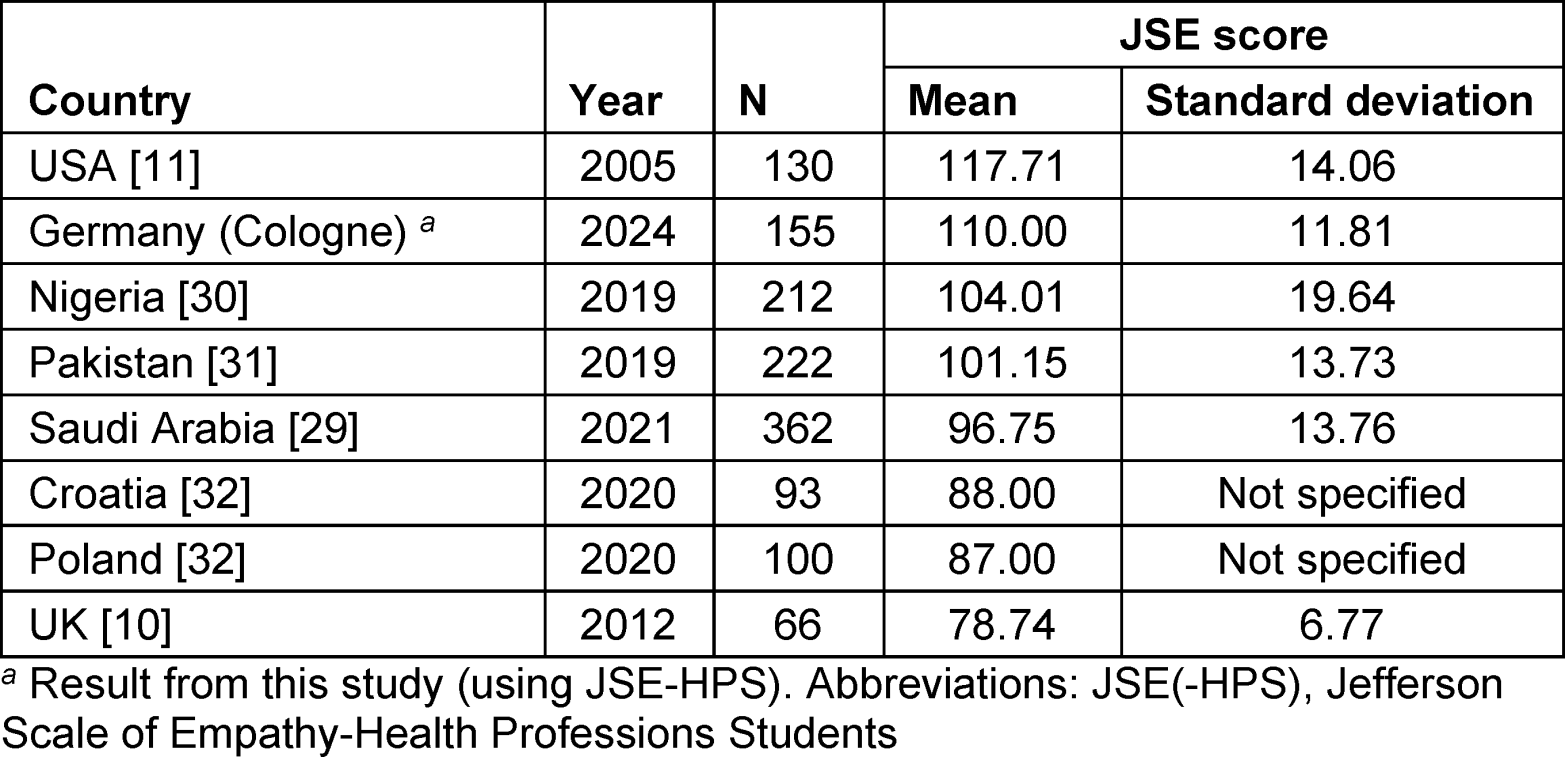
Comparison of mean empathy scores (JSE) among dental students from various countries. Highlighting differences in empathy levels across different educational and cultural contexts
